# Systems and Synthetic Biology of Forest Trees: A Bioengineering Paradigm for Woody Biomass Feedstocks

**DOI:** 10.3389/fpls.2019.00775

**Published:** 2019-06-20

**Authors:** Alexander A. Myburg, Steven G. Hussey, Jack P. Wang, Nathaniel R. Street, Eshchar Mizrachi

**Affiliations:** ^1^Department of Biochemistry, Genetics and Microbiology, Forestry and Agricultural Biotechnology Institute (FABI), University of Pretoria, Hatfield, South Africa; ^2^Forest Biotechnology Group, Department of Forestry and Environmental Resources, North Carolina State University, Raleigh, NC, United States; ^3^Umeå Plant Science Center, Department of Plant Physiology, Umeå University, Umeå, Sweden

**Keywords:** synthetic biology (synbio), systems biology, systems genetics, woody biomass, biorefinery, bioeconomy, lignin biosynthesis, wood formation

## Abstract

Fast-growing forest plantations are sustainable feedstocks of plant biomass that can serve as alternatives to fossil carbon resources for materials, chemicals, and energy. Their ability to efficiently harvest light energy and carbon from the atmosphere and sequester this into metabolic precursors for lignocellulosic biopolymers and a wide range of plant specialized metabolites make them excellent biochemical production platforms and living biorefineries. Their large sizes have facilitated multi-omics analyses and systems modeling of key biological processes such as lignin biosynthesis in trees. High-throughput ‘omics’ approaches have also been applied in segregating tree populations where genetic variation creates abundant genetic perturbations of system components allowing construction of systems genetics models linking genes and pathways to complex trait variation. With this information in hand, it is now possible to start using synthetic biology and genome editing techniques in a bioengineering approach based on a deeper understanding and rational design of biological parts, devices, and integrated systems. However, the complexity of the biology and interacting components will require investment in big data informatics, machine learning, and intuitive visualization to fully explore multi-dimensional patterns and identify emergent properties of biological systems. Predictive systems models could be tested rapidly through high-throughput synthetic biology approaches and multigene editing. Such a bioengineering paradigm, together with accelerated genomic breeding, will be crucial for the development of a new generation of woody biorefinery crops.

## Introduction

Compared to herbaceous plants, forest trees afford numerous of advantages to plant biologists interested in studying growth and development. Most obviously, trees produce vast quantities of wood comprising multiple cells types produced from the meristematic cambial initials of the vascular cambium. In the context of developmental studies of secondary growth, greater size represents greater spatial resolution to profile stages of development during the formation of secondary phloem and xylem as recently demonstrated for aspen (Obudulu et al., 2016; Sundell et al., 2017) and Norway spruce (Jokipii-Lukkari et al., 2017). Such studies have been instrumental in identifying the individual genes, proteins, metabolites, and pathways comprising the molecular components of biological processes such as wood formation. Many wood formation genes have been targeted using transgenic approaches to demonstrate the ability to modify wood properties (Chang et al., 2018). Unfortunately, compared to greenhouse results, some of these single gene modifications have had adverse effects on growth or altered transgene effect when tested in field trials (Leplé et al., 2007; Voelker et al., 2010). Such outcomes suggest a need for greater understanding of the interaction of systems components with intrinsic and extrinsic factors that differ between the greenhouse and field (Beckers et al., 2016).

In the context of industrial application, woody biomass is a renewable, carbon-neutral source of lignocellulosic materials for construction, pulp, bioenergy and, increasingly, for advanced biomaterials such as nanocellulose (Kunaver et al., 2016; Thomas et al., 2018). These and other biorefinery products, including novel biopolymers and biochemicals, are key to the emerging plant-based bio (Vanholme et al., 2013a; Van de Wouwer et al., 2018). Most woody biomass traits of commercial value are genetically complex. As such, it is challenging to devise strategies determining which genes contribute to these complex phenotypes and to associate these with causal mechanisms. In contrast to genomic selection that can be used as a “black box” route to improve complex traits, bioengineering approaches require systems-level understanding to elucidate the molecular basis of complex traits. “Multi-omics” analyses, together with extensive transgenic perturbation of a biological pathway and mathematical modeling of pathway dynamics, have recently been successfully applied to generate the most detailed systems biology model yet, of lignin biosynthesis, in a forest tree (Wang et al., 2018). The large amount of genetic diversity retained in tree breeding programs has also facilitated the use of natural variation to dissect complex traits (Mizrachi and Myburg, 2016; Mizrachi et al., 2017). Such systems genetics approaches pose opportunities for rapid advances in genomic breeding and provide diverse genetic backgrounds for genetic engineering of biomass traits.

Synthetic biology (“SynBio”) aims to reduce biologically complex systems into discrete functional components that can be combined in numerous ways to create efficient and novel biological products or properties (for a synopsis of SynBio development, see Cameron et al., 2014). This transdisciplinary field draws on engineering principles and standardization of the fabrication process (i.e., gene circuit assembly) (Heinemann and Panke, 2006). Not only can synthetic biology incorporate existing genetic components (biological “parts”) across the three Domains of life, but chemical gene and whole-genome synthesis, directed evolution, model-informed protein engineering, and synthetic regulatory machinery design enable the creation of xenobiological systems. The long-lived nature of trees and their vast reserves of carbon-rich sink tissues present both a challenge and opportunity for accumulating and storing synthetically produced compounds (Wilkerson et al., 2014; Mottiar et al., 2016). Synthetically modified trees could serve as renewable factories for the production of large quantities of custom-designed biomass and biochemicals that do not exist naturally in trees.

We present a brief review of recent progress and our perspective on the application of systems biology approaches to understand complex tree biology, from molecular to organismal level crucial for sustainable production of woody biomass. We propose a bioengineering paradigm based on rational design, drawing on systems-level understanding of biological processes – and the application of synthetic biology and genome editing technologies – to engineer such processes in combination with accelerated, genome-assisted breeding of forest trees.

## Systems Biology: Integrative Understanding of Biological Processes in Forest Trees

**Figure 1:**
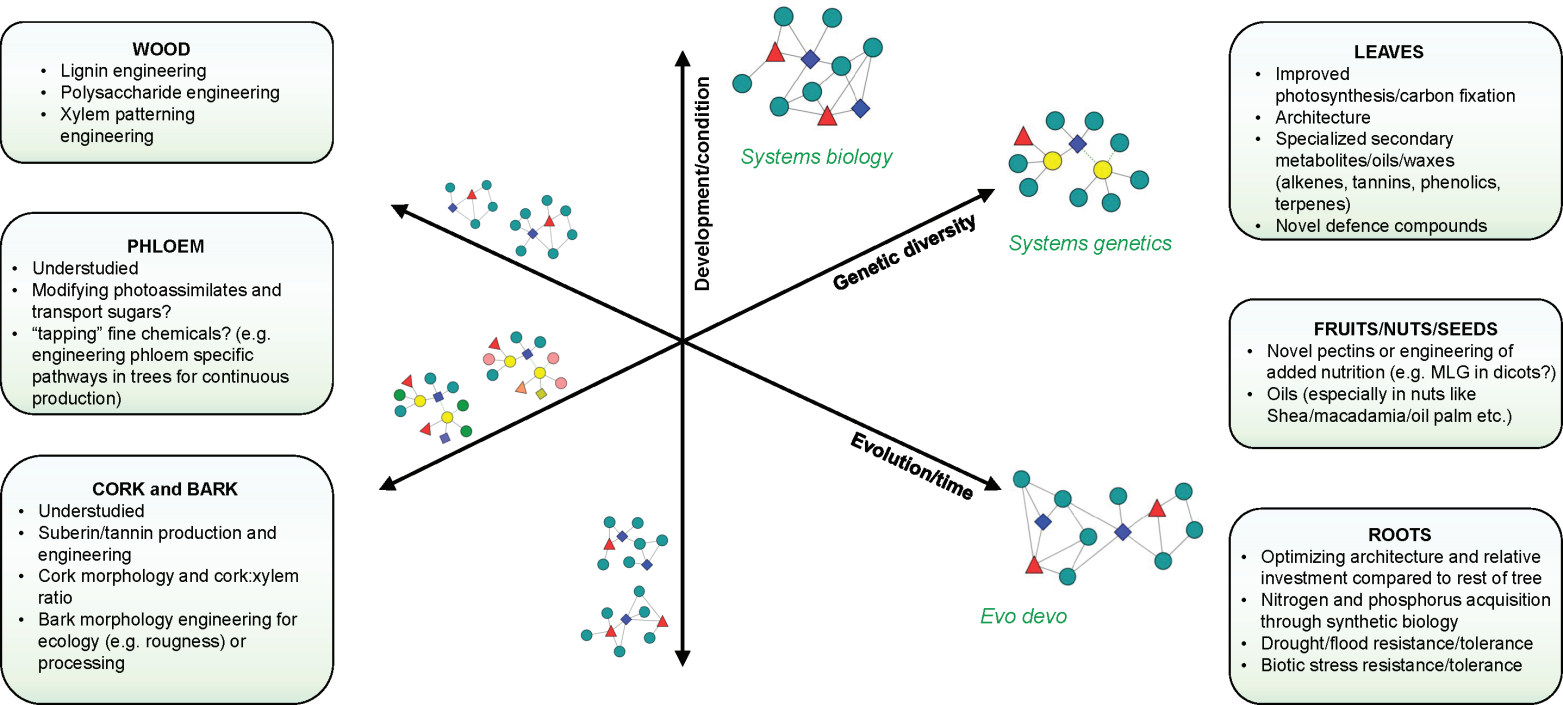
Systems approaches to study biological processes in trees. Axes represent different dimensions or types of perturbation that can be used to dissect biological processes. Network components (colored nodes and shapes) represent the study of different biological layers (DNA, RNA, protein, metabolite, etc.) using integrative approaches that take into account development/condition (*Systems biology*), genetic diversity (*Systems genetics*), and evolution (*Evo-devo*). Yellow nodes in “systems genetics” indicate the connection to complex traits that can be established in population-wide studies. Networks on the left represent intermediate integration steps of components that vary along these axes. Networks on the right represent integrated networks that can be constructed from each type of perturbation. Boxes show target traits and processes associated with different tissues or organs where systems approaches may provide new opportunities for research.

Systems modeling of biological processes requires extensive perturbation of systems components to produce the comprehensive experimental data required for modeling (Figure 1). The lack of extensive mutant collections in forest trees has necessitated the use of transgenic approaches to perturb genes. This has limited such studies to a relatively small number of genes, typically in the same biological pathway. Lignin biosynthesis has emerged as one of the most well-studied biological processes in forest trees, largely due to the interest in reducing lignin content or altering lignin composition to reduce the recalcitrance of woody biomass during industrial processing (Pilate et al., 2002; Leplé et al., 2007; Voelker et al., 2010; Mansfield et al., 2012; Bryan et al., 2016; Saleme et al., 2017). The first comprehensive, systems-level analysis of the lignin biosynthetic pathway was performed in *Arabidopsis* (Vanholme et al., 2012). This resulted in the discovery of additional components of the biological process, in particular, a novel component in the form of a caffeoyl shikimate esterase (CSE) that hydrolyzes caffeoyl shikimate into caffeate and, when mutated, leads to a fourfold increase in glucose release from cellulose during saccharification without pretreatment in *Arabidopsis* (Vanholme et al., 2013b). In poplar downregulated for CSE, 60% more glucose was released from wood without pretreatment, as a consequence of the lower lignin amount and the higher cellulose content (Saleme et al., 2017).

The most extensive analysis of systems biology in forest trees has been done on the lignin biosynthetic pathway in differentiating xylem of *Populus trichocarpa* (Wang et al., 2018). Transgenic trees systematically perturbed in the expression of lignin genes were produced, and quantitative data on genomics, transcriptomics, fluxomics, biochemical, chemical, and cellular analyses were integrated to construct a mathematical description of the pathway. The lignin model calculates how changing expression of any pathway gene or gene combination affects protein abundance, metabolic-flux, and phenotypic traits, including lignin content and composition, tree growth, wood density, and saccharification, for sugar release. The model predicts improvements in any of these traits individually or in combinations, through modifying the expression of specific lignin genes. The lignin pathway is amenable to modeling because major pathway components have been identified and methodologies for their quantification have been developed (Shi et al., 2010; Vanholme et al., 2010, 2019; Wang et al., 2014, 2018). Genomic sequence information (Goujon et al., 2003; Raes et al., 2003; Tuskan et al., 2006; Myburg et al., 2014; Carocha et al., 2015) defined the gene families of the known proteins that are components of the lignin biosynthetic pathway. Some enzymes are encoded by single genes and others are members of large gene families (Goujon et al., 2003; Shi et al., 2010), while other members of the families are not associated with lignification in time or place. This highlights the importance of identifying those family members that are *bona fide* components of the systems model of wood lignification. The abundance of lignin enzymes *in vivo* has been measured in *P. trichocarpa* to determine the quantitative relationship between transcripts and proteins, and the enzymes in the pathway have been purified and characterized biochemically and genetically so reliable kinetics data could be obtained for substrates and inhibitors (Wang et al., 2014).

The systems analysis of lignin biosynthesis in *P. trichocarpa* has provided valuable information on the formation and function of lignin in forest trees, such as where genetic regulation is significantly different from non-woody species (e.g., *Arabidopsis*). For example, in the case of 4-coumarate CoA ligase (4CL), *Arabidopsis* has a single gene and enzyme for 4CL, while *P. trichocarpa* has two distinct *4CL* genes, one encoding a protein with a regulatory function (Chen et al., 2014). Similar regulatory functions have been demonstrated for genes upstream of lignin biosynthesis in the shikimate pathway (Xie et al., 2018) supporting the 4CL finding. *In silico* simulations furthermore predicted the consequence on flux and wood traits for every possible combination of multigene perturbations where expression of lignin genes was either upregulated, downregulated, or remained at the wild-type level. Systems modeling therefore provides a unique opportunity to guide multi-trait engineering and breeding strategies to create novel tree feedstocks optimized for fast growth and conversion to energy and materials. The models also inform identification of alleles or combinations of alleles to breed superior forest tree varieties (Wang et al., 2018).

## Systems Genetics: Dissecting Systems Components and Interactions Underlying Complex Traits

While systems biology studies have advanced our understanding of developmental and stress response pathways (especially in identifying key regulators within these processes), they frequently are based on severe phenotypes that represent an aggregate of a cascade of cellular and physiological reactions to the adverse effect of the perturbation. Furthermore, such studies do not resolve the molecular basis or mechanisms of trait variation among individuals within populations. Revealing how complex trait variation arises from genetic polymorphisms affords opportunities to discover novel approaches to inducing trait variation in addition to advancing understanding of the evolutionary processes and the mechanistic basis of variation in complex traits (Figure 1).

Complex traits are the emergent outcome of actions and interactions of hundreds to thousands of genes, functioning within pathways in combination with environmental effects. Variation at any biological layer from genome to trait can contribute to the final outcome. Full understanding of complex phenotypes requires approaches that can model these individual actions and interactions at the population level. Systems genetics provides a clearer path linking the genome to complex phenotypes by deducing the mechanisms by which genetic variation impacts variation in these intermediate biological layers (e.g., transcript, protein, and/or metabolite) to ultimately impact the complex trait (Civelek and Lusis, 2014; Feltus, 2014). Systems genetics has benefited substantially from recent sequencing technology improvements that have enabled population-wide genome resequencing and global transcript profiling *via* mRNA and small RNA sequencing. While SNP genotyping typically provides adequate genetic resolution for controlled crosses (QTL analysis using pedigrees), genome-wide association studies (GWAS) using natural populations often mandate whole-genome re-sequencing due to the rapid decay of LD in many tree species (McKown et al., 2014; Zhang et al., 2018). There have now been a number of studies in trees mapping the genetic architecture of gene expression variation (eQTLs) in tree pedigrees (Kirst et al., 2004, 2005; Drost et al., 2010) and natural populations (Porth et al., 2013; Mähler et al., 2017; Zhang et al., 2018); however, there is only one reported true systems genetics study (Mizrachi et al., 2017) in addition to genetical genomics approaches (Street et al., 2006; Drost et al., 2015).

These studies have reported contrasting results between pedigrees and natural populations, suggesting that the genetic background is an important consideration, especially for extrapolating findings to a wider context. Due to developments in sequencing technologies, there has been greater focus on eQTL studies; however, variation in protein (allelic) structural variation, epigenomic variation (Cheung et al., 2017), protein abundance (Consoli et al., 2002), and post translational modifications (Cesnik et al., 2016) or metabolome variation (Morreel et al., 2006; Joseph et al., 2014; Matsuda et al., 2015) fit equally well and indeed would be as responsible for variation, within the systems genetics framework. While systems genetics studies can reveal, for example, genomic loci linked to gene expression variation contributing to trait variation, it can be problematic to place these within a biological framework to understand how these novel mechanisms influence the trait. One approach to overcoming this barrier can be integration of systems biology studies, such as developmental studies, to provide context to these novel genes. Combining systems genetics and systems biology co-expression networks is also a powerful approach as biological inference can be aided by considering the neighborhood of genes connected to a novel candidate (Mähler et al., 2017).

To date, one level of complexity that has not been considered in eQTL and systems genetics studies is transcriptional plasticity. There is now extensive knowledge of alternative splicing and transcript usage, which can vary among tissues, during development or among genotypes (Bao et al., 2013; Xu et al., 2014; Zhao et al., 2014). Mapping expression at the transcript level will provide greater resolution to the link between genome and phenotype. However, fine-scale expression analysis at the transcript level remains challenging, mainly because most tissue samples contain a complex mix of transcripts and splicing variants from different cell types or cells at different stages of development. There is equally a need for improvement in network inference methods, with an understanding that no single current method is adequate to capture the range of interactions present in biological networks (Marbach et al., 2012) and with few tools available to facilitate aggregate inference approaches (Schiffthaler et al., 2018). Similarly, no studies have yet integrated genome-wide assays of DNA modifications or accessibility despite increasing evidence of the additional insight such information brings. There is also a paucity of large-scale transcription factor binding or protein-protein interaction data for plants in general, which further limits comprehensive understanding.

## Synthetic Biology: A New Bioengineering Paradigm for Forest Trees

SynBio has made its greatest advancements in prokaryotes and single-celled eukaryotes such as yeast, but plant synthetic biology is catching up (Patron et al., 2015; Schaumberg et al., 2016; de Lange et al., 2018; Hanson and Jez, 2018; Pouvreau et al., 2018). In addition to a large number of modifications made by conventional transgenic approaches (Chang et al., 2018), there have been some notable successes in the synthetic modification of trees using single-gene strategies such as the introduction of chemically labile ester linkages into the lignin backbone of poplar trees through the xylem-specific expression of an exogenous feruloyl-coenzyme A monolignol transferase (FMT) from *Angelica sinensis* (Wilkerson et al., 2014). Future strategies will attempt to evaluate far more complex designs, relying to a large extent on the ability to assemble DNA fragments idempotently (that is, the flexibility to assemble basic parts with increasing complexity using a universal method and without having to re-modify each intermediate). There is currently a scarcity of freely available standardized biological parts suitable for plant biology akin to the International Genetically Engineered Machines (iGEM) BioBrick parts collection^1^. Encouragingly, the Phytobrick synthetic biology standard with a universal lexicon for plant gene elements coupled to powerful Type IIS idempotent assembly methods (Engler et al., 2014; Patron et al., 2015) is fast gaining traction, with an increasing number of Phytobricks now included in the iGEM Standard Registry^2^. Tree biologists and biotechnologists should adapt to this conceptual framework as soon as possible to keep up with synthetic biology development in annual crops. We recently developed an open access synthetic panel of 221 secondary cell wall-related *Eucalyptus grandis* transcription factors and 65 promoter sequences in partnership with the Department of Energy Joint Genome Institute, most of which were designed as Phytobricks (Hussey et al., in preparation). Accessibility of high-throughput DNA synthesis services will ensure that a growing number of standardized parts become available under open material transfer agreements.

One considerable challenge to tree synthetic biology is precise spatiotemporal control of complex multigene constructs, especially in woody tissues where inducing gene expression with external agents is impractical. Such constructs must function optimally in a tissue of interest, be resistant to eukaryotic silencing mechanisms such as RNAi or epigenetic silencing, be somatically stable such that somatic mutations that disrupt a synthetic construct should not be selectively favored, take into account compartmentalized plant cell biology, and have built-in biosafety mechanisms preventing transgene escape. Furthermore, synthetic gene circuits should consist of *composable* parts (de Lange et al., 2018) that individually encode defined and transferrable functions (a property known as *modularity*) and that function independently of endogenous processes to avoid unwanted interference, a property known as *orthogonality*. Designer transcription factors based on zinc finger, TALE, and dCas9 technologies targeting endogenous or synthetic promoters (Liu and Stewart, 2016) are ideal orthogonal synthetic tools that allow considerable transgene regulation flexibility but may require extensive testing and optimization.

**Figure 2:**
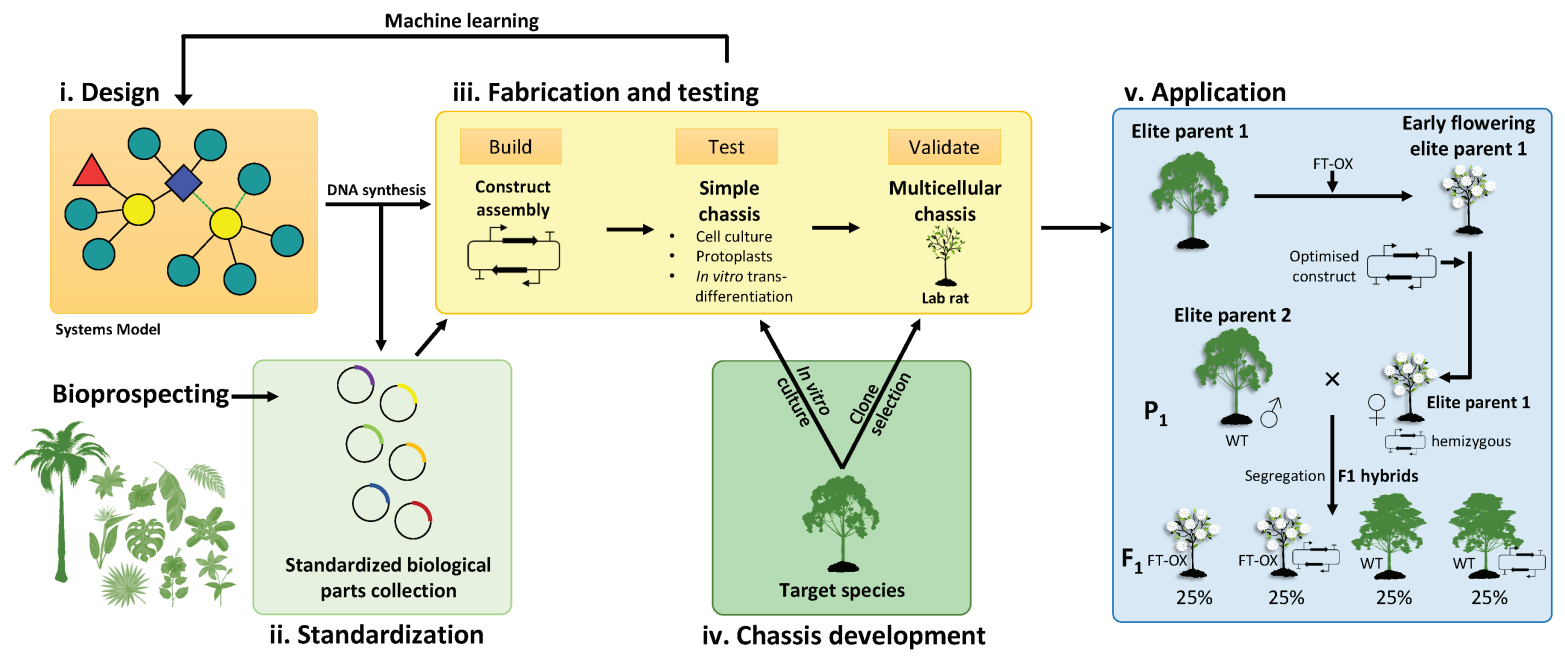
A proposed bioengineering paradigm based on forest tree synthetic biology. **(i)** Systems biology models inform the initial design of synthetic multigene constructs. Individual components (DNA parts) are sourced from existing or novel genetic resources (e.g., bioprospecting), synthesized, and submitted to biological parts collections as standardized Phytobricks **(ii)**. The fabrication and testing phase **(iii)** involves high-throughput idempotent construct assembly followed by transformation and testing in a simplified chassis such as a cell culture derived from the target tree species **(iv)**. Construct expression and phenotypic data are then integrated into a machine learning model to optimize the construct design, an iterative process that produces a reduced number of semi-optimized constructs for validation in a genotype of the target species selected to perform favorably in laboratory conditions and conducive to transformation (“lab rat”). Such validation may include greenhouse trials involving juvenile trees or mature trees in field trials. **(v)** Successful constructs may be introduced into a preferred elite parental genotype for intraspecific or interspecific hybrid breeding. A possible avenue to rapidly mobilize synthetic gene constructs into more diverse genetic backgrounds would be to introduce an early flowering construct (such as overexpression of the *FLOWERING LOCUS T* gene; FT-OX) into a number of elite parental genotypes. Such genotypes can then be transformed with the optimized synthetic gene constructs and used as female parents in crosses with non-transgenic (wild-type, WT) parents to produce F1 progeny segregating for both constructs. If the two constructs are on different chromosomes, approximately 25% of the progeny should be WT for growth and flowering but contain the synthetic gene construct. Early flowering parental genotypes can be propagated *in vitro* to be transformed with various synthetic constructs and, if unrelated, different parental genotypes could be crossed for transgene stacking.

Currently, thousands of iterations of multigene constructs can be produced by robotics-assisted DNA Foundry services. However, it is not feasible to transform and phenotype thousands of transgenic trees for iterative design-build-test-learn cycles envisioned for plant synthetic biology (Pouvreau et al., 2018). Early synthetic designs will therefore have to be tested in a simplified system (or “chassis” in synthetic biology terminology) until optimized constructs can be evaluated in target tree species. This will necessitate the development of experimental chassis derived from the target tree species that are easier to transform and phenotype *en masse*, such as protoplasts, cell cultures, or agroinfiltrated leaves, or “lab rat” genotypes that perform well in tissue culture and have high transformation rates (Figure 2). *In vitro* tracheary element transdifferentiation approaches relying on hormonal induction (Fukuda and Komamine, 1980; Kubo et al., 2005; Saito et al., 2017), or the VND7 inducible system (Yamaguchi et al., 2010; Goué et al., 2013), for example, could be used to induce secondary cell wall formation in suspension culture cells or explant tissues of a target tree species and thus evaluate the phenotypes of many cell wall-modifying constructs before semi-optimized constructs are tested in a multicellular tree model. Successful constructs may either be directly introduced into an elite genotype of the target species or rapidly crossed into elite breeding material after co-transformation of parental genotypes with an early flowering construct (Figure 2).

## Future Perspective

Most fundamental discoveries, including proof-of-concept cell wall and growth modifications (and even extrapolations on biomass processing efficiency), are still derived from the analysis of *Arabidopsis* inflorescence stems, which remains a poor representation of large tree stems comprised mainly of wood. Of priority in the short term is testing genetic perturbations as much as possible in a woody model such as *Populus* and, if possible, directly in target species of interest. Several priorities must be met here, such as enhancing the transformation efficiency of commercial species or genotypes, capacity for large-scale transformation experiments, as well as (crucially) field trials confirming greenhouse phenotypes in mature trees. Large consortia and industry collaborations, as well as engagement and an improvement in the regulatory landscape, must be met for this to be truly realized.

Also in the short to medium term, the convergence of high-resolution technologies that capture genomics, epigenomics, and other cell ‘omics’, phenomics, and environment (including microbiome) data, as well as computational modeling of the interactions of these, requires transdisciplinary innovations and probably the application of artificial intelligence methodology. Combined with genome editing (with broader scale synthetic biology applications), this makes the field of forest biotechnology ripe for a new wave of creativity, especially in thinking of the tree itself as a living biorefinery and as a stable and continuous producer of specialized high-value compounds or polymers in sustainably harvestable tissues and organs such as leaves, secondary phloem, and bark. Higher resolution knowledge of metabolite precursors, tissue-specific pathway engineering, and knowledge of novel high-value derivatives that can be discovered using bioprospecting methods and produced in trees has the potential for a new generation of relatively low volume, but high value, forest products.

How far does the application of these technologies go? Given the long rotation times of forest trees as harvestable biomass crops, it is unlikely (and indeed not essential) that we will see movement toward a “bottom up” approach that builds on a synthetic minimal tree genome. It is much more important to optimize the precise introduction of complex regulatory circuits and metabolic pathways that remain stable through breeding generations, a nascent field of research in itself. Such a bioengineering paradigm, combined with advanced genomic breeding approaches and accelerated flowering technologies, may empower rapid development of woody biomass crops tailored for diverse biorefinery, biomaterials, and timber construction products. In many forest-growing countries, an advanced forest products industry will be one of the cornerstones of the bioeconomy and key to achieving global sustainable development goals.

## Author Contributions

All authors contributed to the drafting and editing of the manuscript.

### Conflict of Interest Statement

The authors declare that the research was conducted in the absence of any commercial or financial relationships that could be construed as a potential conflict of interest.

The handling editor declared a past co-authorship with one of the authors NS.

## Footnotes

^1^http://parts.igem.org

^2^http://parts.igem.org/Collections/Plants
